# Comparative efficacy of various physical activity interventions on individuals with autism spectrum disorder: a systematic review and network meta-analysis

**DOI:** 10.1186/s12889-026-27965-2

**Published:** 2026-06-11

**Authors:** Jun Liu, Jun Sun, Huanyu Li, Jingyu Zhao

**Affiliations:** 1 Schoolof Physical Education and Sports, Central China Normal University, No. 152 LuCRoad, Hongshan District, Wuhan, Hubei 430000 China; 2 School of Physical Education, Wuhan Sports University, No. 461 LuCRoad, Hongshan District, Wuhan, Hubei 430000 China

**Keywords:** Comparative, Various physical activity, Autism spectrum disorder, Social communication, Stereotyped behavior, Motor skill, Network meta-analysis

## Abstract

**Background:**

Physical activity is a promising non-pharmacological intervention for individuals with autism spectrum disorder (ASD), but the most effective intervention regimen remains unclear. This study aimed to compare and rank the effects of different types and doses of physical activity on social communication, stereotyped behaviors, and motor skills in individuals with ASD, thereby providing theoretical guidance for physical activity–based interventions.

**Methods:**

We searched Web of Science, PubMed, Embase, Cochrane Central Register of Controlled Trials, SPORTDiscus, PsycInfo and CNKI from inception to December 2025. Randomized controlled trials, quasi-experimental studies, and controlled studies evaluating the effects of various physical activity interventions on social communication, stereotyped behaviors, or motor skills in individuals with ASD were included. Pairwise analyses and network meta-analyses were conducted using a random effects model.

**Results:**

A total of 45 studies involving 1479 participants were included. The results showed that martial arts had the highest possibility of producing the best effects on social communication (SMD = -0.84, 95% CI : -1.31,-0.37), and team ball sports were most likely to be the most effective in reducing stereotyped behaviors (SMD = -1.39, 95% CI: -2.09,-0.68), while mind-body exercise was identified as the most effective physical activity for improving motor skills (SMD = 2.10, 95% CI: 0.45, 3.75).

Additionally, for social communication, the best effects were observed with a frequency of 3–4 times per week (SMD = -0.90, 95% CI: -1.34, -0.45), session duration of ≤45 minutes (SMD = -0.67, 95% CI: -0.91, -0.43), and an intervention period of more than 12 weeks (SMD = -0.93, 95% CI: -1.18, -0.68). For stereotyped behaviors, the best effects were observed with a frequency of ≥5 times per week (SMD = -1.40, 95% CI: -2.15, -0.65), session duration of ≤45 minutes (SMD = -0.60, 95% CI: -1.10, -0.10), whereas no statistically significant effects were observed for intervention period; For motor skills, the best effects were associated with a frequency of 3–4 times per week (SMD = 2.02, 95% CI: 0.92, 3.12), session duration of＞60 minutes (SMD = 4.35, 95% CI: 1.44,7.26), and a period of ≤8 weeks (SMD = 2.41, 95% CI: 0.90, 3.91).

**Conclusions:**

Martial arts appear to be the most effective intervention for improving social communication, and team ball sports are likely to be the most effective for reducing stereotyped behaviors, whereas mind–body exercises may be the most beneficial for enhancing motor skills in individuals with ASD. In addition, for social communication, the most favorable outcomes were associated with a frequency of 3–4 sessions per week, session duration of ≤45 minutes, and an intervention period of >12 weeks. For stereotyped behaviors, the best effects were observed with a frequency of ≥5 times per week, session duration of ≤45 minutes, however, no statistically significant effects were observed for intervention period. Regarding motor skills, the best effects were found with a frequency of 3–4 times per week, session duration of > 60 minutes, and an intervention period of ≤8 weeks.

**Supplementary Information:**

The online version contains supplementary material available at 10.1186/s12889-026-27965-2.

## Background

Autism spectrum disorder (ASD) is a lifelong neurodevelopmental condition that emerges in early childhood and is characterized by impairments in communication and social interaction, as well as repetitive and stereotyped behaviors [1]. Social communication deficits primarily include difficulties in both verbal and nonverbal communication, atypical conversational patterns, and challenges in understanding others’ thoughts and emotions [1]. Repetitive behaviors refer to high-frequency, inappropriate, and seemingly purposeless actions such as arm-waving, hand-flapping, body-rocking, repetitive object manipulation, and vocal stereotypy [2]. These deficits and behavioral abnormalities often hinder children with ASD from interacting effectively with peers, resulting in social isolation or bullying. Consequently, they may experience reduced self-esteem and self-confidence [3], which can negatively influence their overall development.In addition, approximately 87% of individuals with ASD present with motor difficulties [4], primarily manifested as impairments in coordination of both fine and gross motor skills, balance, flexibility, and gait abilities [5]. Such motor challenges limit their opportunities to participate in daily peer activities [6] and lead to sedentary lifestyles [7], increasing their vulnerability to chronic conditions such as diabetes, obesity, and cardiovascular disease [8].

ASD currently affects more than 78 million people worldwide. According to data from the Autism and Developmental Disabilities Monitoring (ADDM) network, an estimated 1 in 31 eight-year-old children has ASD, and the prevalence continues to rise [9]. A significant amount of time, effort, and resources are invested in the treatment of individuals with ASD, placing a considerable economic burden on families and society [10–12]. Despite long-standing global efforts, no curative pharmacological treatments are available due to the complex pathophysiology of ASD. Existing medications primarily aim to alleviate symptoms [13] and are often accompanied by certain side effects, such as dizziness, somnolence, and reduced appetite [14, 15]. In contrast, non-pharmacological interventions, including Applied Behavior Analysis [16], Denver early intervention model [17] and Floor time [18]-have shown efficacy in improving the core symptoms and associated features of ASD individuals [17–20], and causing virtually no side effects [21]. Consequently, increasing research attention has been directed toward non-pharmacological approaches [16–20]. However, many of these interventions rely on static, desk-based formats and often involve high implementation costs, which together limit their feasibility in flexible or real-world settings [15].

Physical activity has been widely recognized as a non-pharmacological intervention for ASD due to its low cost, ease of implementation, notable effectiveness, and minimal adverse effects [15, 22]. Numerous studies have demonstrated that regular participation in physical activity can improve social interaction and communication skills [23], reduce self-injurious and stereotyped behaviors [24], and enhance motor skills [25] in individuals with ASD. In addition, physical activity has been shown to alleviate negative emotions [26], improve sleep quality [27], and enhance overall quality of life [28].

Currently, various types of physical activity are used as interventions for ASD, primarily including aquatic exercises(AE) [29], fundamental motor skills (FMS) [30], mind-body exercises (MBE) [31], martial arts (MA) [32], team ball sports (TBS) [33], and therapeutic horseback riding(THR) [34], as reported in previous meta-analyses [35, 36]. However, it remains unclear which types of physical activity are most effective for different outcome domains in individuals with ASD. Although several systematic reviews and network meta-analyses have examined the effects of various physical activity interventions, the number and quality of included studies, as well as the range of physical activity types assessed, are still limited. For example, Li et al. included only 8 studies comparing 6 types of physical activity for social communication, and only 5 studies comparing 3 types of physical activity for stereotyped behaviors [35]. One network meta-analysis examined only 3 types of physical activities for reducing stereotyped behaviors [37], whereas a more recent network meta-analysis included only 10 studies [38]. Although one review included a relatively large number and variety of physical activity interventions, the methodological quality of several included studies was limited [39]. These limitations may have affected the reliability of their findings. Furthermore, previous meta-analyses did not investigate the optimal dosage(i.e., frequency, session duration, and intervention period) of physical activity for individuals with ASD.

Existing meta-analyses investigating the optimal dosage of physical activity interventions for individuals with ASD have been constrained by a limited number of available studies, and their conclusions were also inconsistent [15, 37]. Consequently, it remains unclear which specific type and dosage of physical activity are most effective for improving social communication, reducing stereotyped behaviors, or enhancing motor skills in individuals with ASD, as different interventions may benefit different outcome domains. With the continuous publication of new research, incorporating a larger body of evidence into an updated network meta-analysis is essential to generate more robust and up-to-date conclusions.

In summary, this literature review aims to identify the most effective type of physical activity for improving social communication, reducing stereotyped behaviors, and improving motor skills in individuals with ASD, and to explore intervention characteristics such as frequency, session duration, and intervention period by incorporating a large number of studies and synthesizing evidence through network meta-analysis. This review will provide an up-to-date assessment of physical activity interventions for individuals with ASD, offering valuable theoretical insights and practical guidance for future research and application.

## Methods

### Protocol registration and PRISMA guidelines

Our systematic review has been formally registered with the International Prospective Register of Systematic Reviews (PROSPERO) with registration number (CRD420250648822). For the thorough reporting of our work, we have also followed the recommendations of the Preferred Reporting Items for Systematic Reviews and Meta Analyses (PRISMA) [40].

### Literature retrieval strategy

This systematic literature search covered a total of 7 databases, including 6 English-language databases (Web of Science, PubMed, Embase, The Cochrane Library, SPORTDiscus, and PsycInfo) and 1 Chinese-language database(CNKI), encompassing publications from inception to 31 December 2025. The search terms were divided into three components: (1) physical activity interventions (e.g., “physical activity” OR exercise OR sport*); (2) ASD (e.g.“autism spectrum disorder*” OR “autistic spectrum disorder*” OR “autistic disorder*”); (3) Non-ASD conditions (e.g., cancer OR diabetes OR “down syndrome”). The detailed search strategy is provided in Supplementary Appendix 1.

### Literature inclusion and exclusion criteria

Inclusion Criteria: (1) Participants diagnosed with ASD; (2) The primary intervention approach was aquatic exercise, fundamental motor skills, martial arts, mind-body exercise, team ball sports, or therapeutic horseback riding; (3) The control group received routine rehabilitation treatment, no intervention, or was waitlisted; (4) Studies were required to report at least 1 of the following outcoms: social communication, stereotyped behaviors, or motor skills, and provide either raw data or relevant statistical information (e.g.,subgroup data) to allow extraction of physical activity effects, the measurement tools for different outcomes are listed in Supplementary Appendix 2; (5) Eligible study designs included randomized controlled trials(RCT), quasi-experimental studies(QEs), and controlled studies(CTs); (6) Studies were published in English or Chinese and were accessible.

Exclusion Criteria: (1) Studies were excluded if they were not publication or acceptance by a peer-reviewed journal, including books and theses. (2) Reviews, protocols, brief reports, commentaries, dissertations, conference papers and case studies were excluded. (3) Studies involving patients with conditions other than ASD, such as cancer, diabetes, disability, cerebral palsy, down syndrome, traumatic brain injury, etc., were not considered in this review.

### Outcomes

The primary outcomes were social communication deficits and stereotyped behaviors, representing the two core diagnostic domains of ASD. Motor skills performance was considered a secondary outcome, as it is not a core diagnostic feature of ASD but remains an important and commonly observed functional outcome related to physical and developmental performance.

### Literature quality evaluation

Two researchers independently evaluated the quality of all studies included in the review. The methodological quality of the randomized controlled trials was assessed using the Physiotherapy evidence database (PEDro) scale, which consists of 11 items. Each item is scored 1 if the criterion is met and 0 if not, the first item (“eligibility criteria”) is not included in the total score. Accordingly, the total PEDro scale ranges from 0 to 10, with a median score of 5. However, one review have noted that blinding is often infeasible in exercise interventions, making it difficult for such studies to receive points for participant and therapist blinding [41]. Therefore, Considering this inherent limitation of exercise interventions, studies were classified as high quality (≥ 6), moderate quality (4–5), or low quality (≤ 3) based on their PEDro scores [42]. For the non-randomized trials, the Methodological Index for Non-Randomized Studies (MINORS) was employed. For comparative studies, a total of 12 items were assessed, with a score of 0 indicating “not reported”, 1 indicating “reported but inadequate”, and 2 indicating “reported and adequate”, the total score can range from 0 to 24 points. Studies scoring 0–6 points are classified as very low quality, 7–12 points as low quality, 13–18 points as moderate quality, and 19–24 as high quality [43].

### Data extraction

The data extraction process was carried out independently by two researchers using a data extraction form. For English-language articles, data were extracted directly in the original language. For Chinese-language articles, data were extracted in the original language by native Chinese-speaking reviewers without translation. Discrepancies were resolved by discussion or consulting with a third reviewer if necessary. The form included study characteristics(first author’s name, publication year, study design, and location), participant characteristics(sample size, age, and sex), interventions (type, frequency, session duration, intervention period, and comparator information), and outcomes(the relevant statistics used to estimate the effects of physical activity interventions, such as mean, standard deviation, and corresponding measurement tools).

### Statistical analysis

To examine the effects of different types of physical activity on individuals with ASD, a pairwise meta-analysis was first conducted for all outcomes by comparing each intervention with the control group.The random effects model was applied to estimate effect sizes using standardized mean differences (SMD) with 95% confidence intervals (95% CIs) based on post-intervention scores.

Stata 16.0 software was used to conduct network meta-analysis, and effect sizes were estimated with SMDs and 95% CIs. For social communication and stereotyped behaviors, lower SMD values indicate better intervention effects, whereas for motor skills, higher SMD values indicate better intervention effects. In network evidence plot, the size of each node represented the sample size for a given intervention, and the lines connecting the nodes reflected direct comparisons between interventions.The thicker the line, the more studies support the direct comparison between the two interventions, conversely, the thinner the line, the fewer the studies. If a closed loop was present in the network, the global inconsistency test was performed. If *P* > 0.05, it indicates that there is no significant inconsistency between direct and indirect comparisons, and the node splitting method was used for the local inconsistency test. The effectiveness ranking of different physical activity interventions was reported using the surface under the cumulative ranking area (SUCRA) and mean ranks, with larger SUCRA values and smaller mean ranks indicating better intervention performance. In addition, publication bias was assessed by the generated comparison-adjusted funnel plot.

## Results

### Study selection

**Fig. 1:**
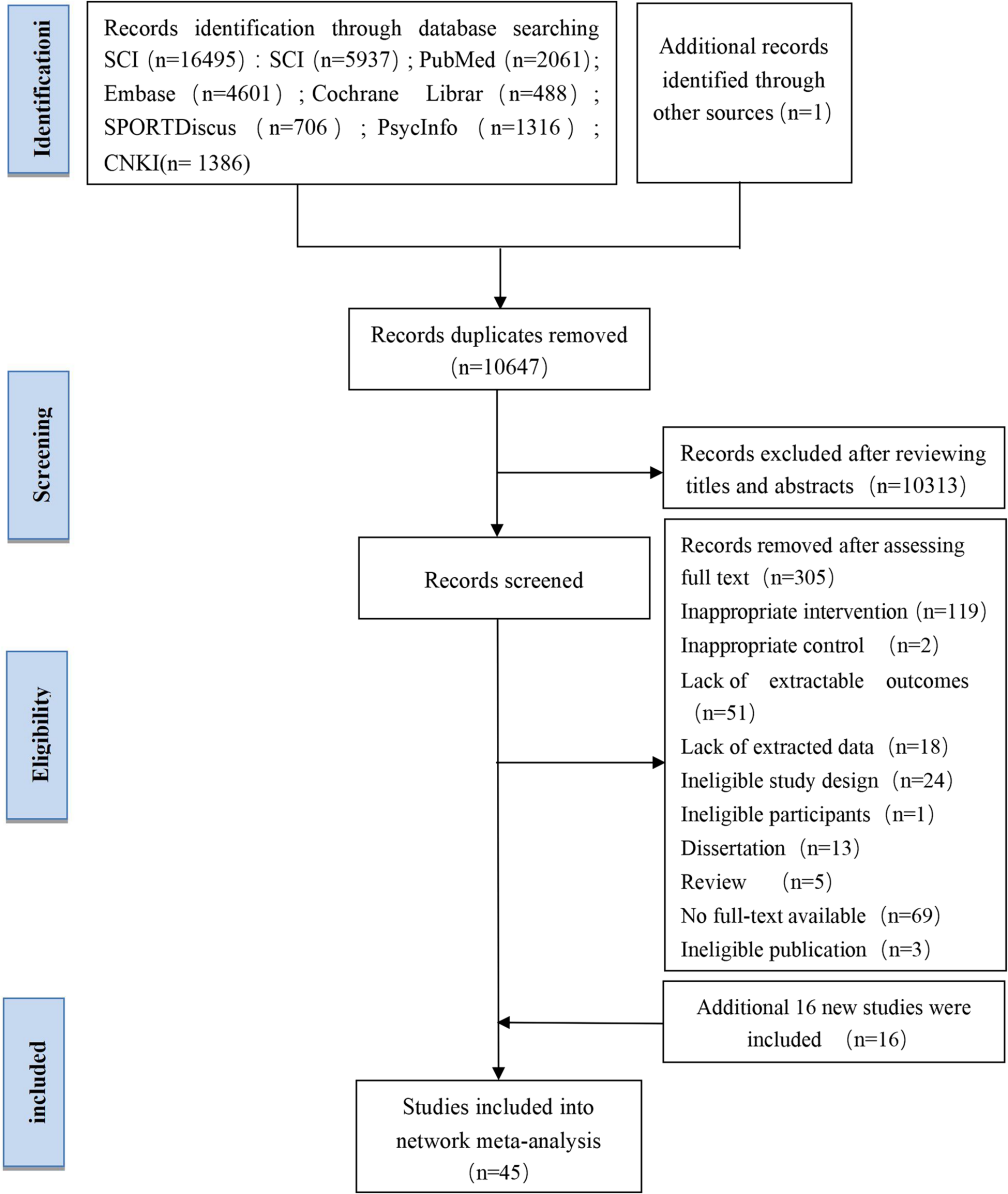
Selection of studies for inclusion

The literature search strategy identified 16,496 potentially eligible studies, including 5937 from Web of Science, 2061 from PubMed, 4601 from Embase, 488 from The Cochrane Library, 706 from SPORTDiscus, 1316 from PsycInfo, 1386 from CNKI, and 1 through other sources. After removing duplicates, titles and abstracts of 10,313 studies were screened, resulting in 334 papers selected for full-text review. Ultimately, 29 studies were included in the network meta-analysis. An updated search (up to November 2025) identified an additional 16 studies meeting the inclusion criteria. Therefore, a total of 45 studies were included in the meta-analysis (Fig. 1).

### Inter-observer agreement

The inter-rater reliability during full-text screening was excellent (k = 0.81). There was minimal disagreement observed between the two independent raters.

### Characteristics of the studies

A total of 1479 participants(775 in the intervention groups and 704 in the controls) were included across the 45 studies. The average age of individuals with ASD ranged from 3.99 to 13.29 years, with 80.3% being male. The studies were published between 2009 and 2025 and conducted in various countries: Australia (*n* = 2), Canada (*n* = 1), China (*n* = 18), Colombia (*n* = 1), India (*n* = 2), Iran (*n* = 10), Italy (*n* = 2), Poland(*n* = 1),Slovakia(*n* = 1), Turkey(*n* = 1), UK(*n* = 2), and USA(*n* = 4). Of the 45 intervention studies included in this review, 29 (64.4%) were randomized controlled trials, 9 (20.0%) were quasi-randomized controlled trials, and 7 (15.6%) were controlled trials.

Regarding intervention categories, the included studies included 6 types of physical activity: aquatic exercise(studies: *n* = 3, participants: *n* = 73), fundamental motor skills (studies: *n* = 10, participants: *n* = 259), martial arts(studies: *n* = 3, participants: *n* = 116), mind-body exercise (studies: *n* = 8, participants: *n* = 217), team ball sports(studies: *n* = 13, participants: *n* = 432), and therapeutic horseback riding(studies: *n* = 6, participants: *n* = 301). In addition, 1 study directly compared the effects of aquatic exercise and martial arts(study: *n* = 1, participants: *n* = 30), and another 1 study directly compared the effects of martial arts and team ball sports(study: *n* = 1, participants: *n* = 51). Intervention frequency ranged from 0.5 to 5 times per week, and the session duration ranged from 15 to 90 min. The intervention length ranged from 4 to 44 weeks, with more than 60% of studies reporting interventions lasted at least 12 weeks. A detailed summary of each included study (*n* = 45) is presented in Appendix 3.

### Methodological quality

Among the randomized trials, 1 studies score 5, indicating “moderate” methodological quality. The remaining studies scored between 6 and 9 (4 scored 6, 21 scored 7, 3 scored 8, and 1 scored 9), all of which were considered high methodological quality. For the non-randomized trials, scores ranged from 13 to 23. Most studies were of moderate methodological quality (scores 13–18: 1 scored 13, 3 scored 14, 6 scored 16, and 3 scored 18), while 2 studies (scores 20 and 23) were classified as high methodological quality. (Appendix 4 ).

### Primary outcomes

**Fig. 2:**
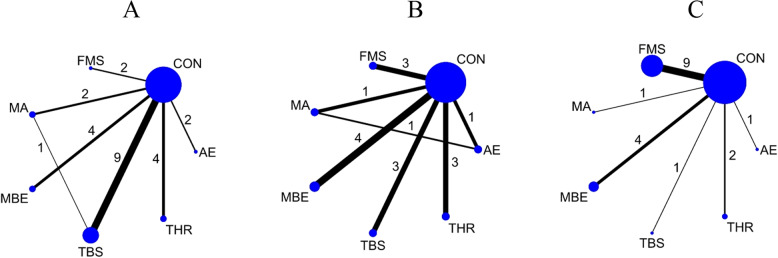
Network plots of physical activity intervention type for **A** social communication, **B** stereotyped behaviors, and **C** motor skills. Notes: AE means Aquatic exercise; FMS means Fundamental motor skills; MA means Martial arts; MBE means Mind-body exercise; TBS means Team ball sports; THR means Therapeutic horseback riding. The numbers on the edges represent the number of studies comparing each pair of interventions

**Fig. 3:**
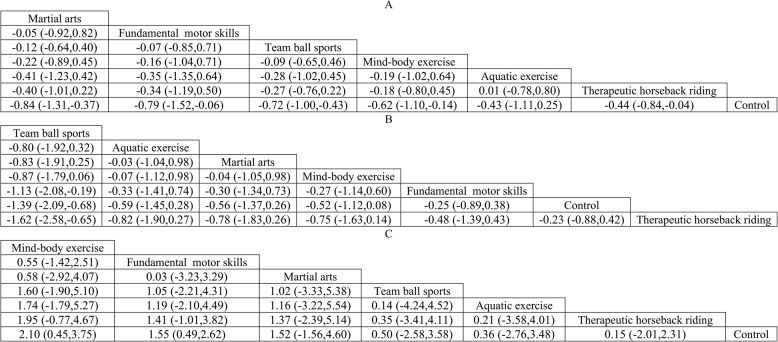
League table of the effects of physical activity intervention type doses on **A** social communication, **B** stereotyped behaviors, and **C** motor skills. Notes. For social communication and stereotyped behaviors, lower SMD values indicate better intervention effects, whereas for motor skills, higher SMD values indicate better intervention effects

**Fig. 4:**
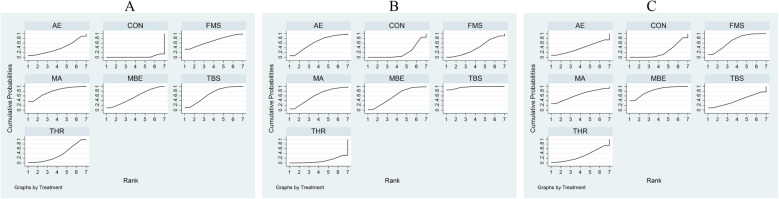
Cumulative ranking probability plots of physical activity intervention type for **A** social communication, **B** stereotyped behaviors, and **C** motor skills. Notes. AE means Aquatic exercise; CON means Control; FMS means Fundamental motor skills; MA means Martial arts; MBE means Mind-body exercise; TBS means Team ball sports; THR means Therapeutic horseback riding

For social communication, a total of 24 studies with 881 participants were included. Among these, 2 studies examined the effects of aquatic exercise [44, 45], 2 studies investigated the effects of fundamental motor skills [23, 46], 2 studies assessed the effects of martial arts [47, 48], 4 studies evaluated the effects of mind-body exercise [49–52], 9 studies focused on team ball sports [33, 53–60], 4 studies examined the effects of therapeutic horseback riding [34, 61–63], and 1 study compared the intervention effects of martial arts and team ball sports [64]. Pairwise meta-analyses compared the efficacy of various physical activity interventions with control groups, and no significant global inconsistency was detected in the network meta-analysis. Detailed results are provided in Supplementary Appendix 5.The network plot of intervention comparisons is presented in Fig. 2A, with only martial arts and team ball sports directly compared. The results of our network meta-analysis indicated that fundamental motor skills (SMD = -0.79,95% CI: -1.52, -0.06), martial arts (SMD = -0.84,95% CI: -1.31, -0.37), mind-body exercise (SMD = -0.62,95% CI: -1.10, -0.14), team ball sports (SMD = -0.72,95% CI: -1.00, -0.43) and therapeutic horseback riding (SMD: -0.44, 95% CI: -0.84,-0.04) were effective (Fig. 3A). Martial arts had the highest probability (35.9%) of being the most intervention, with a surface under the cumulative ranking curve (SUCRA) value of 78.4%(Table 1; Fig. 4A). Furthermore, the results of the comparison-adjusted funnel plot are provided in Supplementary 5, and no significant asymmetry was observed. In addition, the effects of intervention dose on social communication were further analyzed. The most effective interventions for social communication were those conducted 3–4 times a week (SMD = -0.90, 95% CI: -1.34, -0.45), with sessions ≤ 45 min (SMD = -0.67, 95% CI: -0.91, -0.43), and intervention periods longer than 12 weeks (SMD = -0.93, 95% CI: -1.18, -0.68) (Table 2 and Appendix 6).

**Table 1:** The rankings of social communication, stereotyped behaviors and motor skills for different types of physical activity

Physical activity	Social communication			Stereotyped behaviors			Motor skills		
	SUCRA (%)	PrBest(%)	Mean rank	SUCRA (%)	PrBest(%)	Mean rank	SUCRA (%)	PrBest(%)	Mean rank
Control	2.5	0.0	6.9	19.9	0.0	5.8	23.4	0.0	5.6
Aquatic exercise	39.6	7.6	4.6	62.4	6.5	3.3	39.1	8.7	4.7
Fundamental motor skills	69.0	34.5	2.9	40.8	0.5	4.6	69.2	11.6	2.8
Martial arts	78.4	35.9	2.3	60.7	5.2	3.4	64.2	27.9	3.1
Mind-body exercise	56.3	10.0	3.6	59.5	2.0	3.4	81.8	39.7	2.1
Team ball sports	67.0	10.7	3.0	96.8	85.8	1.2	41.5	9.8	4.5
Therapeutic horseback riding	37.3	1.3	4.8	9.8	0.0	6.4	30.8	2.3	5.2

Higher SUCRA values and lower mean ranks indicate better-performing physical activity intervention. Probability of it being the best physical activity intervention (PrBest)

For stereotyped behaviors, a total of 16 studies were eligible for network meta-analysis, including 532 participants. Among these, 1 study examining the effects of aquatic exercise [65], 3 studies investigating the effects of fundamental motor skills [46, 66, 67], 1 study examined the effects of martial arts [68], 4 studies assessed the effects of mind-body exercise [50–52, 69], 3 studies investigated the effects of team ball sports [24, 70, 71], 3 studies examined the effects of therapeutic horseback riding [34, 61, 72], and 1 study compared the intervention effects of aquatic exercise and martial arts [73]. Pairwise meta-analyses compared the efficacy of various physical activity interventions with control groups, and no significant global inconsistency was detected in the network meta-analysis; detailed results are presented in Supplementary Appendix 5. The network plot for stereotyped behaviors (Fig. 2B) shows all available comparisons from the included trials, with only aquatic exercise and martial arts being directly compared. The comparative effects among various physical activity interventions are presented in Fig. 3.B. Rankings based on cumulative probability plots and SUCRAs values are shown in Table 1; Fig. 4B. The results of the SUCRA revealed that only team ball sports exerted a significant effect (SMD=-1.39, 95% CI:-2.09,-0.68) on reducing stereotyped behaviors, with a SUCRA value of 96.8% (Table 2). The comparison-adjusted funnel plot did not show obvious asymmetry, indicating no clear evidence of publication bias(Supplementary 5 ). For stereotyped behaviors, the most effective interventions were those conducted at least 5 times per week (SMD = -1.40, 95% CI: -2.15, -0.65), with sessions ≤ 45 min (SMD = -0.60, 95% CI: -1.10, -0.10), whereas, no optimal intervention period was identified (Table 2 and Appendix 6).

**Table 2:** Physical activity dose analysis

Intervention characteristics		k	ES(g)	[95% Conf. Interval]	*p*
Social communication					
Frequency (times/week)	1–2	10	-0.63	-0.90,-0.36	0.000
	3–4	1	-0.90	-1.34,-0.45	0.000
	≥ 5	6	-0.63	-1.00,-0.26	0.001
Session duration /minutes	≤ 45	15	-0.67	-0.91,-0.43	0.000
	46–60	6	-0.62	-0.97,-0.28	0.000
	> 60	2	-0.58	-1.15,-0.01	0.047
Period/week	≤ 8	1	0.11	-0.90,0.68	0.788
	9–12	14	-0.52	-0.69,-0.36	0.000
	>12	5	-0.93	-1.18,-0.68	0.000
Stereotyped behaviors					
Frequency (times/week)	1–2	6	-0.49	-1.02,0.03	0.066
	3–4	4	-0.31	-0.93,0.30	0.317
	≥ 5	3	-1.40	-2.15,-0.65	0.000
Session duration/minutes	≤ 45	9	-0.60	-1.10,-0.10	0.019
	46–60	6	-0.27	-0.88,0.33	0.375
Period/week	≤ 8	3	-0.14	-1.14,0.86	0.784
	9–12	11	-0.32	-0.82,0.17	0.203
	>12	2	-0.85	-2.02,0.32	0.155
Motor skills					
Frequency (times/week)	1–2	7	1.11	-0.03,2.25	0.056
	3–4	8	2.02	0.92,3.12	0.000
	≥ 5	1	0.5	-2.42,3.42	0.737
Session duration/minutes	≤ 45	8	0.66	-0.26,1.58	0.162
	46–60	9	1.55	0.66,2.44	0.001
	> 60	1	4.35	1.44,7.26	0.003
Period/week	≤ 8	4	2.41	0.90,3.91	0.002
	9–12	11	1.17	0.29,2.04	0.009
	>12	3	0.40	-1.24,2.04	0.634

95% CI means confidence intervals (lower limit, upper limit); ES(g) means effect size(Std diff in means); k means the number of studies

### Secondary outcomes

For motor skills, 18 studies including 610 participants were included. Among these, 1 studies examined the effects of aquatic exercise [44], 9 studies investigated the effects of fundamental motor skills [23, 30, 46, 67, 74–78], 1 studies investigated the effects of martial arts [48], 4 studies assessed the effects of mind-body exercise [50, 79–81], 1 studies assessed the effects of team ball sports [25], and 2 studies examined the effects of therapeutic horseback riding [82, 83]. Pairwise meta-analyses were conducted to compare the efficacy of various physical activity interventions with the control groups(Supplementary Appendix 5). The network plot is shown in Fig. 2C. Comparisons among various physical activity interventions and control groups (Fig. 3C) suggest that fundamental motor skills(SMD = 1.55, 95% CI:0.49,2.62) and mind-body exercise(SMD = 2.10, 95% CI:0.45,3.75) exerted a significant effect on motor skills. Mind-body exercise had the highest probability (39.7%) of being the most effective intervention, with a SUCRA value of 81.8% (Table 1; Fig. 4C). The comparison-adjusted funnel plot did not reveal substantial asymmetry, suggesting no clear evidence of publication bias(Supplementary 5). The best effects for motor skills were observed with interventions conducted 3–4times a week (SMD = 2.02, 95% CI: 0.92, 3.12), session duration of more than 60 min (SMD = 4.35, 95% CI: 1.44, 7.26), and intervention period of ≤ 8 weeks (SMD = 2.41, 95% CI: 0.90, 3.91) (Table 2 and Appendix 6).

## Discussion

The objective of this comprehensive literature review was to compare the effects of various physical activities on individuals with ASD. To the best of our knowledge, this is the first and one of the most extensive largest review examining the effects of different types and dosages of physical activity interventions on social communication, stereotyped behaviors, and motor skills in individuals with ASD. Our findings suggest that martial arts have the highest probability of being the most effective physical activity for improving social communication, team ball sports were most likely to reduce stereotyped behaviors, while mind-body exercises may be the most effective for enhancing motor skills in individuals with ASD. Furthermore, this study reveals that different dosages of physical activity are associated with varying intervention effects for different outcomes.

The results of this study suggest that martial arts may be the most effective type of physical activity for improving social communication among individuals with ASD. This effect may be related to the structured and rule-based nature of martial arts interventions. According to Albert Bandura’s social learning theory, key elements such as modeling, imitation, and timely feedback in group-based or organized physical activities can significantly facilitate the development of social skills, especially for children with ASD who have limited opportunities for natural social learning [33]. In martial arts interventions, children with ASD engage in repetitive practice, action imitation, and peer interaction, which may strengthen observational learning and behavioral internalization, thereby improving social deficits. Second, martial arts emphasize social etiquette, which can enhance social skills. Interactions with instructors and peers in a structured environment allow children to practice social behaviors [84]. For example, karate classes often begin with etiquette training, such as bowing and greetings, which teach respect and basic social norms. These skills can then be generalized to daily life for children with ASD. Given the pronounced social communication difficulties in this population, many martial arts interventions incorporate structured opportunities for social engagement, such as partner exercises and team-based tasks, to help children attend to others, understand intentions, improve communication and cooperation, and develop a sense of teamwork. Another distinctive feature of martial arts interventions is their ability to improve coordination and balance [85]. Training typically includes movements such as kicking, punching, and defensive actions, which enhance muscle strength, flexibility, and reaction speed. Improvements in these physical abilities may increase self-confidence, thereby encouraging children with ASD to engage more actively in social interactions. Furthermore, some researchers have suggested that martial arts training may increase levels of Brain-Derived Neurotrophic Factor, promoting neuroplasticity and thereby enhancing learning and skill acquisition, which in turn may improve a range of abilities, including communication skills [32]. The auditory reception–motor execution processes involved in martial arts instructions may also activate neural connections between language-related regions in the left hemisphere and the motor cortex [86]. During partner training, eye contact and coordinated movements may contribute to the development of prefrontal social cognitive networks [87]. Such cross-regional neural coordination may help simultaneously enhance social, motor, and communication abilities. It is worth noting that, although this study found no beneficial effects of aquatic exercise on social communication, this result may be due to the limited number of available studies. In this review, only 2 study investigated the effects of aquatic exercise. Therefore, these findings should not be considered definitive, and further research is needed to explore the intervention effects of this types of physical activity.

The results of this study suggest that team ball sports may be the most effective type of physical activity for reducing stereotyped behaviors in individuals with ASD. This finding is consistent with another study, although that study only compared the effects of three types of physical activity on stereotypical behaviors in this population [37]. Previous research has shown that stereotyped behaviors in individuals with ASD are primarily driven by the need to seek or escape sensory stimuli and to maintain homeostasis [88]. The physical movements involved in team ball sports—such as running, jumping, jogging, throwing, catching, and kicking—are similar to those observed in stereotyped behaviors. These comparable movements may provide similar sensory feedback and pleasurable experiences, potentially substituting for stereotyped behaviors and reducing their occurrence [68]. Additionally, fatigue resulting from participation in team ball sports could further contribute to the reduction of stereotyped behaviors. In our review, team ball sports were performed at the highest frequency among all physical activity types, reaching up to 5 sessions per week. High-frequency participation is more likely to induce fatigue, which in turn may help decrease stereotyped behaviors [24].

Our results indicate that mind–body exercise may be the most effective type of physical activity for enhancing motor skills in individuals with ASD. The positive effects of mind-body exercise on motor skills have been confirmed by numerous studies [69, 79, 89]. In the studies included in this review, mind–body exercise produced substantial improvements in motor skills; for example, scores increased from 5.94 to 11.48 [81], and from 40.83 to 71 [79]. Mind–body exercise may improve motor skills through multiple mechanisms. Individuals with ASD often exhibit deficits in cerebellar function, which plays a critical role in motor control. These deficits include damage to Purkinje and granule neurons, as well as abnormalities in the posterior hemisphere and vermis, which are linked to bidirectional pathways between the visual, auditory, and somatosensory cortices [90]. Mind–body exercise can effectively stimulate the cerebellum and help ameliorate these deficiencies. These exercises typically guide participants to practice in a calm, focused state, redirecting attention from distracting external stimuli to their own body movements [91]. Unlike other types of physical activity that specifcally emphasize awareness of body movements [92], mind–body exercise emphasizes the coordination of breathing, body awareness, and movement execution during different asanas or exercises. This approach engages the visual, auditory, and proprioceptive systems, promoting sensory integration and improving motor skills [93]. Sensory integration training can also enhance balance by increasing activity in the standing limb muscles and trunk extensors, thereby improving stability and motor performance [94]. In addition, mind–body exercise can facilitate the synchronous enhancement of memory and electroencephalogram signals, improve cortical adaptability for motor learning, and enhance neuromuscular coordination, ultimately leading to improved motor skills in individuals with ASD [95].

This study also analyzed the effects of different intervention frequency, session duration, and intervention period of physical activity on social communication, stereotyped behaviors and motor skills in individuals with ASD. In terms of the frequency of physical activity, the optimal intervention frequency for improving social and motor skills was consistent, at 3–4 times per week, whereas reducing stereotyped behaviors required a higher intervention frequency(≥ 5 times per week).This discrepancy may be related to the type of physical activity, as our review found that only team ball sports performed 5 times per week had a beneficial effect on reducing stereotyped behaviors. Future research should comprehensively consider the influence of each component of a physical activity intervention program, such as the type, frequency, session duration, and period on stereotyped behaviors in individuals with ASD, in order to design more effective interventions. Regarding session duration, social communication and stereotyped behaviors showed consistent findings, with an optimal duration of ≤ 45 min per session. This suggests that, for these two core symptoms, longer intervention time is not necessarily more beneficial; rather, a moderate duration may yield the best effects. Although the optimal duration for motor skills appeared to be > 60 min, this estimate was based on only 1 study and therefore remains inconclusive. Regarding the intervention period, no consistency was observed across the three outcomes in this study, as different indicators showed divergent results. Surprisingly, the effects of different intervention periods on motor skills decreased as the intervention period increased, which was contrary to the findings for the other two indicators. This may be linked to the varying difficulty levels of physical activities in improving each specific outcome. This suggests that for the different symptoms of ASD individuals, targeted physical activity intervention plans of different doses should be set up to achieve the best intervention effect on them.

The results of our network meta-analysis differ from those of several previous reviews. For example, Li’s network meta-analysis shows that fundamental motor skills may have the best effect on social communication, while kata techniques appeared to be most effective in reducing stereotyped behavior in individuals with ASD [35]. This discrepancy may be attributed to the smaller number of studies included in Li’s review. Specifically, only 8 studies were analyzed for social communication, resulting in just 1 study being included for 5 of the 6 physical activity interventions examined. In contrast, our study included a total of 24 studies to compare the effects of 6 types of physical activity on social communication, all 6 types represented by 2 or more studies, thereby enhancing the reliability of our conclusions. Another network meta-analysis found that combination exercise were the most effective type of physical activity for improving social communication in ASD individuals [39]. There are two possible reasons for the discrepancy with our study. First, the limited quality of the included studies may have influenced the results. As noted in their review, many of the included studies were of lower quality, which could have affected the analysis of combination exercises. In contrast, our study ensured the reliability of conclusions by applying stricter inclusion criteria, particularly regarding study quality. For studies published in Chinese, only those indexed in the Chinese Social Sciences Citation Index (CSSCI) or its extended version were included. Second, the relatively small number of studies in previous reviews may have contributed to the differences. For example, their review included only 4 studies on team ball sports, whereas our review included 9. The number of included studies can substantially impact the accuracy of network meta-analysis results. 

### Strengths and limitations

#### Strengths

First, our review included a substantial number of high-quality studies, including the most recently published ones, which greatly increased both the quantity and quality of evidence compared to previous reviews. To our knowledge, this is the largest network meta-analysis to explore the efficacy of various physical activity interventions on social communication, stereotyped behaviors, and motor skills in individuals with ASD, allowing for more reliable conclusions by incorporating a greater number of studies. Second, by including a large number of studies, we were able to analyze the optimal intervention dose for physical activity in this population, providing up-to-date evidence for designing effective interventions.

#### limitations

First, this review included only studies published in English and Chinese. Although this approach helped capture relevant studies published in both English and Chinese, the exclusion of publications in other languages may introduce potential language bias and limit the generalizability of the findings. Secondly, some intervention nodes, such as martial arts, aquatic exercise, therapeutic horseback riding, and fundamental motor skill training, were supported by only 1 or 2 studies, which may affect the stability of indirect comparisons and SUCRA-based rankings to some extent. Additionally, when comparing the intervention effects of a single element, we cannot rule out the influence of other factors, as physical activity intervention programs consist of multiple components, including type, frequency, session duration, and period. Finally, differences in the age, sex, and disease severity in the study samples may also influence the results.

## Conclusion

Our network meta-analysis, by synthesizing a large body of evidence from previous studies, examined the effects of different physical activities on individuals with ASD. This provides valuable insights for physical activity interventions in individuals with ASD. Our findings suggest that martial arts have the highest likelihood of being the most effective physical activity for improving social communication, team ball sports appear most effective for reducing stereotyped behaviors, while mind-body exercises may be most effective for enhancing motor skills in individuals with ASD. Furthermore, this study highlights that different dosages of physical activity yield varying intervention effects on these outcome measures. However, given the limitations of our meta-analysis, the results should be interpreted with caution. Future research should focus on conducting more multi-group randomized controlled trials to provide stronger evidence regarding the relative efficacy of various exercise interventions.

## Supplementary Information

Supplementary Material 1.

## Data Availability

The authors confirm that the data supporting the findings of this study are available in the original published studies. In addition, the corresponding author may provide additional information if reasonably requested.

## Abbreviations

ADDM: Autism and Developmental Disabilities Monitoring
AE: Aquatic exercises
ASD: Autism spectrum disorder
CI: Confidence interval
CSSCI: Chinese Social Sciences Citation Index
CT: Controlled studies
ES: Effect size
FMS: Fundamental motor skills
MA: Martial arts
MBE: Mind-body exercises
MINORS: Methodological index for non-randomized studies
PEDro: Physiotherapy Evidence Database
PRISMA: Preferred Reporting Items for Systematic Reviews and Meta Analyses
PROSPERO: Prospective Register of Systematic Reviews
QE: Quasi-experimental study
RCT: Randomized controlled trials
SMD: Standardized mean differences
SUCRA: Surface under cumulative ranking curve
TBS: Team ball sports
THR: Therapeutic horseback riding
