# Supplementary material for: Comparative efficacy of various physical activity interventions on individuals with autism spectrum disorder: a systematic review and network meta-analysis
Source: BMC Public Health. 2026 Jun 11;26:2377. doi: 10.1186/s12889-026-27965-2 (PMC13479576; doi:10.1186/s12889-026-27965-2)
Supplement: Supplementary file 1 [file 12889_2026_27965_MOESM1_ESM.docx]

Contents of Supplementary materials

[Appendix 1 Search strategy	2](#_Toc30220)

[Appendix 2 The measurement tools for different outcomes	3](#_Toc18381)

[Appendix 3 Study characteristics	4](#_Toc26516)

[Appendix 4 Methodological quality	1](#_Toc14838)0

[Appendix 5 Effects of different types of physical activity on ASD outcomes	1](#_Toc3947)3

[Appendix 6 Effects of different doses of physical activity on ASD outcomes](#_Toc1788)	23

Appendix 7 PRISMA 2020 Checklist	38

# **Appendix 1 Search strategy**

Table 1 Search Strategy for English Databases: Web of Science Example

| Step | Search strategy |
| --- | --- |
| #1 | ((TS=(“physical activity” OR exercise OR sport* OR “horseback riding” OR horse OR equine OR football OR basketball OR volleyball OR ball OR yoga OR “tai chi” OR “nei yang gong” OR dance OR “mind–body” OR aquatic OR water OR swim OR karate OR kata OR Judo OR “martial arts” OR “motor skill*”)) |
| #2 | TS=(“autism spectrum disorder*” OR “autistic spectrum disorder*” OR “autistic disorder*” OR “autistic spectrum*” OR “autism spectrum” OR autis* OR ASD)) |
| #3 | TI=(cancer OR diabetes OR “down syndrome” OR “cerebral palsy” OR “traumatic brain injury” OR “multiple sclerosis” OR “attention deficit hyperactivity disorder” OR ADHD OR “learning disability” OR disability OR meta OR review OR case) |
| #4 | #1 AND #2 NOT #3 |

Table 2 Chinese search strategy

| Step | Search strategy |
| --- | --- |
| #1 | （篇关摘：身体活动 + 运动 + 骑马 + 足球 + 篮球 + 排球 + 球 + 瑜伽 + 太极 + 内养功 + 舞蹈 + 身心运动 + 游泳 + 水 + 空手道 + 柔道 + 武术 + 动技能(精确)） |
| #2 | （篇关摘：自闭症 + 孤独症(精确)） |
| #3 | #1 AND #2 |

# **Appendix 2 The measurement tools for different outcomes**

Table 2 The measurement tools for different outcomes

| *outcomes* | *measurement tools* |
| --- | --- |
| Social Communication | Social Responsiveness Scale (SRS);Vineland Adaptive Behavior Scale-third edition(VABS-3);Social Skills Improvement System;Social Skills Improvement System Rating Scales(SSIS);social communication questionnaire(SCQ);(Gilliam Autistic Disorder Rating Scale‑2‑TV,GARS-2-TV);communication subscale of Gilliam Autism Rating Scale-Second Edition(CSGARS-2); |
| Stereotyped behavior | Aberrant Behavior Checklist(ABC);Gilliam autism rating scale(GARS-2);The Social Responsiveness Scale-Second Edition(SRS-2);Repetitive Behavior Scale-Revised(RBS-R);Gilliam Autistic Disorder Rating Scale-2-TV(GARS-2-TV);Autism Diagnosis Observation Scale(ADOS); |
| Motor Skills | Test of Gross Motor Development Second Edition(TGMD-2);Brininx-Oresetsky Test (BOT);Peabody Developmental Motor Scales-2(PDMS-2);The Movement Assessment Battery for Children(M-ABC-2);Vineland Adaptive Behavior Scale(VABS);the third version of the Abbreviated Development Scale (EAD-3);Gross motor function measure (GMFM)； |

**Appendix 3 Study characteristics**

Table 3 Characteristic of included studies

| Study | | |  | Participants (Intervention vs control) | | |  | Interventions | | | comparator | Outcomes |
| --- | --- | --- | --- | --- | --- | --- | --- | --- | --- | --- | --- | --- |
| The first author,year | Country | Design |  | Sample size | Age  (years) | Male  /Female |  | duration | frequency | Length |  |  |
| **Aquatic exercise** | | | | | |  |  |  |  |  |  |  |
| AdibSaber,2024 | Iran | QE |  | 20(10/10) | 10.70 ± 1.88 vs  10.40 ± 2.06 | NR |  | 60 | 2 | 10 | Routine rehabilitation | Stereotyped behaviors |
| Caputo,2018 | Italy | CT |  | 26(13/13) | 8.30 ± 2.30 vs  7.70 ± 2.00 | 11/2 vs 6/7 |  | 45 | 1~2 | 40 | Routine rehabilitation | Social communication  Motor skills |
| Marzouki,2022 | Iran | RCT |  | 14(8/6) | 6.30 ± 0.50 vs  6.30 ± 0.50 | 7/1 vs 5/1 |  | 50 | 2 | 8 | Daily activities | Stereotyped behaviors |
|  |  |  |  | 14(8/6) | 6.40 ± 0.50 vs  6.30 ± 0.50 | 8/0 vs 5/1 |  | 50 | 2 | 8 |  |  |
| Zanobin,2019 | Italy | CT |  | 25(13/12) | 5.69 ± 1.27 vs  5.52 ± 1.54 | 10/3 vs 9/3 |  | 30 | 0.5 | 24 | Daily activities | Social communication |
| **Fundamental motor skills** | | | | | | | | | | | |  |
| Arabi,2019 | Iran | RCT |  | 30(15/15) | 8.40 ± 2.01 vs  8.44 ± 1.94 | 12/3 vs 11/4 |  | 60 | 3 | 10 | Routine rehabilitation | Stereotyped behaviors  Motor skills |
| Arabi,2025 | Iran | QE |  | 30(15/15) | 8.59 ± 2.12 vs  8.44 ± 1.44 | 10/5 vs 11/4 |  | 45 | 3 | 12 | Routine rehabilitation | Motor skills |
| Bremer,2015 | Canada | RCT |  | 9(5/4) | - | - |  | 60 | 1 | 12 | wait-list | Motor skills |
| Castaño,2023 | Colombia | RCT |  | 20(10/10) | 5.80 ± 1.39 vs  5.80 ± 1.61 | 8/2 vs 9/1 |  | 60 | 3 | 8 | Routine rehabilitation | Motor skills |
| The first  author,year | Country | Design |  | Sample size | Age  (years) | Male  /Female |  | duration | frequency | Length |  |  |
| Felzer-Kim,2020 | USA | RCT |  | 14(8/6) | 4.49 ± 0.58 vs  4.49 ± 0.60 | 7/1 vs 3/3 |  | 15 | 4 | 10 | Routine rehabilitation | Motor skills |
| Hassani,2020 | Iran | RCT |  | 19(10/9) | 9.10±0.87 vs  8.70 ± 0.70 | 6/4 vs 6/3 |  | 60 | 2 | 8 | Non-intervention | Motor skills |
|  |  |  |  | 20(11/9) | 8.55±0.68 vs  8.70 ± 0.70 | 8/3 vs 6/3 |  | 60 | 2 | 8 |  |  |
| Hatipoğlu Özcan,2024 | Turkey | CT |  | 34(17/17) | 4.84±0.73 vs  4.69 ± 0.75 | 16/1vs 16/1 |  | 60 | 2 | 12 | Routine rehabilitation | Social communication  Stereotyped behaviors  Motor skills |
| Kaplanova,2023 | Slovakia | CT |  | 20(10/10) | 6.78±1.34 vs  8.25±1.44 | 9/1 vs 8/2 |  | 30 | 2 | 8 | No sports activities | Motor skills |
| Moradi,2020 | Iran | RCT |  | 50(25/25) | 7.62±1.15 | 25/0 vs 25/0 |  | 60 | 3 | 12 | Non-intervention | Stereotyped behaviors |
| Wang,2025 | China | RCT |  | 22(12/10) | 7.75 ± 1.65 vs  7.63 ± 1.58 | 11/1 vs 9/1 |  | 45 | 4 | 18 | Daily activities | Social communication  Motor skills |
| **Martial arts** | | | | | | | | | | | |  |
| AdibSaber,2024 | Iran | QE |  | 20(10/10) | 10.6 ± 2.06 vs  10.4± 2.06 | NR |  | 60 | 2 | 10 | Routine rehabilitation | Stereotyped behaviors |
| Bahrami,2012 | Iran | RCT |  | 30(15/15) | 9.20 ± 3.32 vs  9.06 ± 3.33 | 13/2 vs 13/2 |  | 30~90 | 4 | 14 | No sports activities | Stereotyped behaviors |
| Bahrami,2016 | Iran | RCT |  | 30(15/15) | 9.20 ± 3.32 vs  9.06 ± 3.33 | 13/2 vs 13/2 |  | 30~90 | 4 | 14 | Routine rehabilitation | Social communication |
| Study | | |  | Participants ( Intervention vs control) | | |  | Interventions | | | comparator | Outcomes |
|  | | |  |  | | |  |  | | |  |  |
| The first  author,year | Country | Design |  | Sample size | Age  (years) | Male  /Female |  | duration | frequency | Length |  |  |
| Fu,2025 | China | RCT |  | 56(28/28) | 6.18 ± 0.61 vs  5.93 ± 0.77 | 18/10vs13/15 |  | 60 | 3 | 24 | Routine rehabilitation | Social communication  Motor skills |
| Jafari,2025 | Iran | RCT |  | 34(17/17) | 4.07 ± 0.43 vs  4.13 ± 0.45 | - |  | 60 | 3 | 12 | Usual care | Social communication |
| **Mind-body exercise** | | | | | | | | | | | |  |
| Aithal,2021 | UK | RCT |  | 26(10/16) | 11.53 vs 9.77 | 21vs5 |  | 40 | 2 | 5 | Usual care | Social communication |
| Cui,2024 | China | RCT |  | 24(12/12) | 8.08 ± 1.97 vs  8.25±1.28 | 12/0 vs 12/0 |  | 80 | 3 | 12 | Daily activities | Motor skills |
| Ju,2024 | China | RCT |  | 17(9/8) | 11.11 ± 2.52 vs  12.75 ± 2.31 | 6/3 vs 6/2 |  | 40~45 | 3 | 8 | Daily activities | Stereotyped behaviors |
| Lindor,2023 | Australia | RCT |  | 20(8/12) | 9.00 ± 1.47 vs  9.08 ± 1.44 | - |  | 60 | 1 | 10 | Wait list | Social communication  Stereotyped behaviors  Motor skills |
| Ma,2025 | China | QE |  | 28(17/11) | 13.29 ± 1.80 vs  13.18 ± 1.60 | 12/5 vs 10/1 |  | 40 | 2 | 44 | Routine rehabilitation | Social communication  Stereotyped behaviors |
| Sarabzadeh,2019 | Iran | RCT |  | 18(9/9) | 8.88 ± 1.76 vs  8.22 ± 1.92 | 7/2 vs 7/2 |  | 60 | 3 | 6 | Non-intervention | Motor skills |
| Shanker,2022 | India | RCT |  | 41(23/18) | - | - |  | 45 | NR | 12 | Daily activities | Motor skills |
| Study | | |  | Participants (Intervention vs control) | | |  | Interventions | | | comparator | Outcomes |
| The first  author,year | Country | Design |  | Sample size | Age  (years) | Male  /Female |  | duration | frequency | Length |  |  |
| Shanker,2023 | India | RCT |  | 43(23/20) | 9.77 ± 2.63 vs  9.61 ± 1.93 | 19/4 vs 16/4 |  | 45 | NR | 12 | Daily activities | Social communication  Stereotyped behaviors |
| **Team ball sports** | | | | | | | | | | | |  |
| Cai,2020,a | China | QE |  | 29(15/14) | 5.13 ± 0.61 vs  4.68 ± 0.72 | 12/3 vs 13/1 |  | 40 | 5 | 12 | Daily activities | Social communication |
| Cai,2020,b | China | QE |  | 30(15/15) | 5.03 ± 0.64 vs  4.56 ± 0.84 | 12/3 vs 14/1 |  | 40 | 5 | 12 | Daily activities | Social communication |
| Dong,2020 | China | RCT |  | 30(15/15) | 4.67 ± 0.70 vs  4.97 ± 0.61 | 12/3 vs 13/2 |  | 40 | 5 | 12 | Routine rehabilitation | Stereotyped behaviors |
| Howells,2020 | Australia | CT |  | 40(19/21) | 7.98 ±1.71 vs  8.62 ± 2.26 | 17/2 vs 20/1 |  | 60~90 | 1 | 12 | Routine rehabilitation | Social communication |
| Jafari,2025 | Iran | RCT |  | 34(17/17) | 3.99 ± 0.43 vs  4.13 ± 0.45 | - |  | 60 | 3 | 12 | Usual care | Social communication |
| Qi,2024 | China | RCT |  | 28(14/14) | 5.04±0.66 vs  4.71±0.75 | 12/2 vs 13/1 |  | 40 | 5 | 12 | Routine rehabilitation | Social communication |
| Qi,2025 | Poland | RCT |  | 43(23/20) | 6.52 ± 2.19 vs  6.40 ± 2.30 | 20/3 vs 18/2 |  | 45 | 5 | 12 | Routine rehabilitation | Motor skills |
| Qiao,2024 | China | RCT |  | 34(19/15) | 5.13±0.64 vs  4.67±0.69 | 16/3 vs 13/2 |  | 40 | 5 | 12 | Routine rehabilitation | Social communication |
| SP,2023 | China | RCT |  | 36(18/18) | 9.89 ±2.19 vs  10.39±2.40 | 16/2 vs 14/4 |  | 40 | 2 | 16 | Routine rehabilitation | Social communication |
| Study | | |  | Participants (Intervention vs control) | | |  | Interventions | | | comparator | Outcomes |
| The first  author,year | Country | Design |  | Sample size | Age  (years) | Male  /Female |  | duration | frequency | Length |  |  |
|  |  |  |  |  |  |  |  |  |  |  |  |  |
| Wang,2020 | China | QE |  | 33(18/15) | 5.11 ± 0.65 vs  4.70 ± 0.70 | 15/3 vs 13/2 |  | 40 | 5 | 12 | Daily activities | Stereotyped behaviors |
| Yang,2021 | China | QE |  | 30(15/15) | 4.67 ± 0.70 vs  5.03±0.55 | 13/2 vs 12/3 |  | 40 | 5 | 12 | Routine rehabilitation | Social communication |
| Yang,2024 | China | QE |  | 30(15/15) | 5.17 ± 0.72 vs  4.67 ± 0.7 | 13/2 vs 13/2 |  | 40 | 5 | 12 | Routine rehabilitation | Social communication |
| Zhang,2025 | China | RCT |  | 28(15/13) | 4.90 ± 0.66 vs  4.77 ± 0.70 | - |  | 40 | 5 | 12 | Routine rehabilitation | Stereotyped behaviors |
| Zhao,2018 | China | QE |  | 41(21/20) | 6.14±0.96 vs  6.10±0.98 | 14/7 vs 15/5 |  | 60 | 2 | 12 | waiting list | Social communication |
| **Therapeutic horseback riding** | | | | | | | | | | | |  |
| Coman,2018 | USA | RCT |  | 50(25/25) | 8.84±1.72 vs  8.56±1.5 | 19/6 vs 23/2 |  | 70 | 1 | 12 | waiting list | Social communication |
| Gabriels,2015 | USA | RCT |  | 97(50/47) | - | - |  | 45 | NR | 10 | Non-intervention | Social communication  Stereotyped behaviors  Motor skills |
| Harris,2017 | UK | QE |  | 24(10/14) | 7.96±0.78 vs  6.97±0.33 | 9/1 vs 12/2 |  | 45 | NR | 7 | No intervention | Stereotyped behaviors |
| Pan,2019 | USA | RCT |  | 16(8/8) | 11.88 ± 2.45 vs  9.8 ± 2.82 | 6/2 vs 7/1 |  | 45 | 1 | 10 | No intervention | Social communication  Stereotyped behaviors |
| Zhao,2022 | China | RCT |  | 53(26/27) | 6.00±1.70 vs  5.80±1.50 | 15/11 vs 18/9 |  | 60 | 2 | 12 | No intervention | Motor skills |
| Study | | |  | Participants (Intervention vs control) | | |  | Interventions | | | comparator | Outcomes |
| The first  author,year | Country | Design |  | Sample size | Age  (years) | Male  /Female |  | duration | frequency | Length |  |  |
| Zhao,2021 | China | QE |  | 61(31/30) | 7.06±1.50 vs  7.13±1.36 | 21/10 vs  23/7 |  | 60 | 2 | 16 | Routine rehabilitation | Social communication |

Note: “-”indicates that the value is missing, CT, controlled trial; NR, not reported, QE, quasi-experimental, RCT, randomized controlled trial;

Note: RCT, randomized controlled trial; CT, controlled trial; NR, not reported;

**Appendix 4：Methodological quality**

Table 4 Physiotherapy evidence database (PEDro) scale ratings

| Study | Item1 | Item2 | Item3 | Item4 | Item5 | Item6 | Item7 | Item8 | Item9 | Item10 | Item11 | Total |
| --- | --- | --- | --- | --- | --- | --- | --- | --- | --- | --- | --- | --- |
| Aithal,2021 | 1 | 1 | 0 | 1 | 0 | 0 | 0 | 1 | 1 | 1 | 1 | 7 |
| Arabi,2019 | 1 | 1 | 0 | 1 | 0 | 0 | 0 | 1 | 1 | 1 | 1 | 7 |
| Bahrami,2012 | 1 | 1 | 0 | 1 | 0 | 0 | 0 | 1 | 1 | 1 | 1 | 7 |
| Bahrami,2016 | 1 | 1 | 0 | 1 | 0 | 0 | 0 | 1 | 1 | 1 | 1 | 7 |
| Bremer,2014 | 1 | 1 | 0 | 1 | 0 | 0 | 0 | 1 | 1 | 1 | 1 | 7 |
| Castaño,2023 | 1 | 1 | 0 | 1 | 0 | 0 | 0 | 1 | 1 | 1 | 1 | 7 |
| Coman,2018 | 1 | 1 | 0 | 1 | 0 | 0 | 0 | 1 | 1 | 1 | 1 | 7 |
| Cui,2024 | 1 | 1 | 0 | 1 | 0 | 0 | 0 | 1 | 1 | 1 | 1 | 7 |
| Dong,2020 | 1 | 1 | 0 | 1 | 0 | 0 | 0 | 1 | 1 | 1 | 1 | 7 |
| Felzer-Kim,2020 | 0 | 1 | 0 | 1 | 0 | 0 | 0 | 1 | 1 | 1 | 1 | 6 |
| Fu,2025 | 1 | 1 | 0 | 1 | 0 | 0 | 1 | 1 | 1 | 1 | 1 | 8 |
| Gabriels,2015 | 1 | 1 | 0 | 1 | 0 | 0 | 0 | 0 | 1 | 1 | 1 | 6 |
| Hassani,2020 | 1 | 1 | 0 | 1 | 0 | 0 | 0 | 1 | 1 | 1 | 1 | 7 |
| Hatipoğlu Özcan,2024 | 1 | 1 | 0 | 1 | 0 | 0 | 0 | 1 | 1 | 1 | 1 | 7 |
| Jafari,2025 | 1 | 1 | 1 | 1 | 0 | 0 | 1 | 1 | 1 | 1 | 1 | 9 |
| Ju,2024 | 1 | 1 | 1 | 1 | 0 | 0 | 0 | 1 | 1 | 1 | 1 | 8 |
| Lindor,2023 | 1 | 1 | 0 | 1 | 0 | 0 | 0 | 0 | 1 | 1 | 1 | 6 |
| Marzouki,2022 | 1 | 1 | 0 | 1 | 0 | 0 | 0 | 1 | 1 | 1 | 1 | 7 |
| Moradi,2020 | 1 | 1 | 0 | 1 | 0 | 0 | 0 | 1 | 1 | 1 | 1 | 7 |
| Pan,2019 | 1 | 1 | 0 | 1 | 0 | 0 | 0 | 1 | 1 | 1 | 1 | 7 |
| Qi,2024 | 1 | 1 | 0 | 1 | 0 | 0 | 1 | 1 | 1 | 1 | 1 | 8 |
| Qi,2025 | 0 | 1 | 0 | 1 | 0 | 0 | 0 | 0 | 1 | 1 | 1 | 5 |

| Study | Item1 | Item2 | Item3 | Item4 | Item5 | Item6 | Item7 | Item8 | Item9 | Item10 | Item11 | Total |
| --- | --- | --- | --- | --- | --- | --- | --- | --- | --- | --- | --- | --- |

| Qiao,2024 | 1 | 1 | 0 | 1 | 0 | 0 | 0 | 1 | 1 | 1 | 1 | 7 |
| --- | --- | --- | --- | --- | --- | --- | --- | --- | --- | --- | --- | --- |
| Sarabzadeh,2019 | 1 | 1 | 0 | 1 | 0 | 0 | 0 | 1 | 1 | 1 | 1 | 7 |
| Sha,2023 | 1 | 1 | 0 | 1 | 0 | 0 | 0 | 1 | 1 | 1 | 1 | 7 |
| Shanker,2022 | 1 | 1 | 0 | 1 | 0 | 0 | 0 | 1 | 1 | 1 | 1 | 7 |
| Shanker,2023 | 1 | 1 | 0 | 1 | 0 | 0 | 0 | 1 | 1 | 1 | 1 | 7 |
| Wang,2025 | 1 | 1 | 0 | 1 | 0 | 0 | 0 | 1 | 1 | 1 | 1 | 7 |
| Zhang,2025 | 1 | 1 | 0 | 1 | 0 | 0 | 0 | 0 | 1 | 1 | 1 | 6 |
| Zhao,2022 | 1 | 1 | 0 | 1 | 0 | 0 | 0 | 1 | 1 | 1 | 1 | 7 |

Note: Item1, eligibility criteria; Item 2, random allocation; Item 3, concealed allocation; Item 4, baseline comparability; Item 5, blind subjects; Item 6, blind therapists; Item 7, blind assessors; Item 8, adequate follow-up; Item 9, intention-to-treat analysis; Item 10, between-group comparisons; Item 11, estimates and variability.

Table 5 Methodological index for non-randomized studies (MINORS).

| Study | Item1 | Item2 | Item3 | Item4 | Item5 | Item6 | Item7 | Item8 | Item9 | Item10 | Item11 | Item12 | Total |
| --- | --- | --- | --- | --- | --- | --- | --- | --- | --- | --- | --- | --- | --- |
| AdibSaber,2024 | 2 | 2 | 2 | 2 | 0 | 0 | 0 | 0 | 2 | 2 | 2 | 2 | 16 |
| Arabi,2025 | 2 | 2 | 2 | 2 | 2 | 2 | 2 | 2 | 2 | 2 | 2 | 2 | 23 |
| Cai,2020,a | 2 | 2 | 2 | 2 | 0 | 0 | 0 | 0 | 2 | 2 | 2 | 2 | 16 |
| Cai,2020,b | 2 | 2 | 2 | 2 | 0 | 0 | 0 | 0 | 2 | 2 | 2 | 2 | 16 |
| Caputo,2018 | 2 | 2 | 2 | 2 | 2 | 0 | 0 | 0 | 2 | 2 | 2 | 2 | 18 |
| Harris,2017 | 2 | 2 | 2 | 2 | 0 | 0 | 0 | 0 | 0 | 2 | 2 | 2 | 14 |
| Howells,2020 | 2 | 2 | 2 | 2 | 0 | 2 | 2 | 0 | 2 | 2 | 2 | 2 | 20 |
| Kaplanova,2023 | 2 | 2 | 2 | 2 | 0 | 0 | 0 | 0 | 2 | 2 | 2 | 2 | 16 |
| Ma,2025 | 2 | 1 | 2 | 2 | 0 | 0 | 0 | 0 | 0 | 2 | 2 | 2 | 13 |
| Wang,2020 | 2 | 2 | 2 | 2 | 0 | 0 | 0 | 2 | 2 | 2 | 2 | 2 | 18 |
| Yang,2021 | 2 | 2 | 2 | 2 | 0 | 0 | 0 | 0 | 2 | 2 | 2 | 2 | 16 |
| Yang,2024 | 2 | 2 | 2 | 2 | 0 | 0 | 0 | 2 | 2 | 2 | 2 | 2 | 18 |
| Zanobin,2019 | 2 | 2 | 2 | 2 | 0 | 0 | 0 | 0 | 2 | 2 | 2 | 2 | 16 |
| Zhao,2018 | 2 | 2 | 2 | 2 | 0 | 0 | 0 | 0 | 0 | 2 | 2 | 2 | 14 |
| Zhao,2021 | 2 | 2 | 2 | 2 | 0 | 0 | 0 | 0 | 0 | 2 | 2 | 2 | 14 |

Note: Item1, A clearly study aimed; Item2, Inclusion of consecutive patients; Item3: Prospective collection of data; Item4: Endpoints appropriate to the aim of the study; Item5: Unbiased assessment of the study endpoint; Item6: Follow-up period appropriate to the aim of the study; Item7: Loss to follow less than 5%; Item8: Prospective calculation of the study size; Item9: An adequate control group; Item10: Contemporary groups; Item11: Baseline equivalence of groups;Item12. Adequate statistical analyses

**Appendix 5 Effects of different types of physical activity on ASD outcomes**

Figure 1 pairwise analysis for social communication

***Global inconsistency test***

χ^2^ =0.64 *p* =0.7249

No evidence for the existence of significant global inconsistency

***Local inconsistency test***

Figure 2 Loop-specific heterogeneity for social communication

Note. A means control; B means aquatic exercise E means Martial arts

Figure 3 pairwise analysis for stereotyped behaviors

***Global inconsistency test***

χ^2^ =1.20, *p* =0.5482

No evidence for the existence of significant global inconsistency

***Local inconsistency test***

Figure 4 Loop-specific heterogeneity for stereotyped behaviors

Note. A means control; B means aquatic exercise E means martial arts

Figure 5 pairwise analysis for motor skills

Figure 6 Interval plot of network meta-analysis for social communication

in ASD individuals

Abbreviations: AE means Aquatic exercise; FMS means Fundamental motor skills; MA means Martial arts; MBE means Mind-body exercise; TBS means Team ball sports; THR means Therapeutic horseback riding 4.21

Figure 7 Interval plot of network meta-analysis for stereotyped behavior

in ASD individuals

Abbreviations: AE means Aquatic exercise; FMS means Fundamental motor skills; MA means Martial arts; MBE means Mind-body exercise; TBS means Team ball sports; THR means Therapeutic horseback riding

Figure 8 Interval plot of network meta-analysis for motor skill in ASD individuals

Abbreviations: AE means Aquatic exercise; FMS means Fundamental motor skills; MA means Martial arts; MBE means Mind-body exercise; TBS means Team ball sports; THR means Therapeutic horseback riding

Social Communication	Stereotyped Behaviors	Motor Skills

Figure 9 Forest plots of physical activity intervention type for each ASD outcome

*Note*.A means Control group; B means Aquatic exercise; C means Fundamental motor skills; D means Martial arts; E means Mind-body exercise; F means Team ball sports; G means Therapeutic horseback riding

Social Communication	Stereotyped Behaviors	Motor Skills

Figure 10 Comparison-adjusted funnel plots of physical activity intervention type for each ASD outcome

*Note*.A means Control group; B means Aquatic exercise; C means Fundamental motor skills; D means Martial arts; E means Mind-body exercise; F means Team ball sports; G means Therapeutic horseback riding

**Appendix 6 Effects of different doses of physical activity on ASD outcomes**

Social Communication	Stereotyped Behaviors	Motor Skills

Figure 11 Network plots of physical activity intervention frequencies for each ASD outcome

*Notes*. The numbers on the edges represent the number of studies comparing each pair of interventions.

Social Communication	Stereotyped Behaviors	Motor Skills

Figure 12 Network plots of physical activity intervention session durations for each ASD outcome

*Notes*. The numbers on the edges represent the number of studies comparing each pair of interventions.

Social Communication	Stereotyped Behaviors	Motor Skills

Figure 13 Network plots of physical activity intervention periods for each ASD outcome

*Notes.* The numbers on the edges represent the number of studies comparing each pair of interventions.

Social Communication	Stereotyped Behaviors	Motor Skills

Figure 14 Forest plots of physical activity intervention frequencies for each ASD outcome

*Note*. A means control group; B means 1–2 times/week; C means 3–4 times/week; D means ≥5 times/week

Social Communication	Stereotyped Behaviors	Motor Skills

Figure 15 Forest plots of physical activity intervention session durations for each ASD outcome

*Note*. A means control group; B means ≤45 minutes; C means 46–60 minutes; D means＞ 60 minutes

Social Communication	Stereotyped Behaviors	Motor Skills

Figure 16 Forest plots of physical activity intervention periods for each ASD outcome

*Note*. A means control group; B means ≤8 weeks; C means 9–12 weeks; D means ＞12weeks

Social Communication	Stereotyped Behaviors	Motor Skills

Figure 17 Cumulative ranking probability plots of physical activity intervention frequencies for each ASD outcome

Social Communication	Stereotyped Behaviors	Motor Skills

Figure 18 Cumulative ranking probability plot of physical activity intervention session durations for each ASD outcome

Social Communication	Stereotyped Behaviors	Motor Skills

Figure 19 Cumulative ranking probability plots of physical activity intervention periods for each ASD outcome

Social Communication	Stereotyped Behaviors	Motor Skills

Figure 20 Comparison-adjusted funnel plots of physical activity intervention frequencies for each ASD outcome

*Note*. A means control group; B means 1–2 times/week; C means 3–4 times/week; D means ≥5 times/week

Social Communication	Stereotyped Behaviors	Motor Skills

Figure 21 Comparison-adjusted funnel plots of the effects of physical activity intervention session durations for each ASD outcome

*Note*. A means control group; B means ≤45minutes; C means 46–60minutes; D means＞60minutes

Social Communication	Stereotyped Behaviors	Motor Skills

Figure 22 Comparison-adjusted funnel plots of physical activity intervention periods for each ASD outcome

*Note*. A means control group; B means ≤8 weeks; C means 9–12weeks; D means＞12weeks

A

| 3–4 times/week |  |  |  |
| --- | --- | --- | --- |
| -0.26 (-0.84,0.32) | ≥5 times/week |  |  |
| -0.27 (-0.79,0.26) | -0.00 (-0.46,0.46) | 1–2 times/week |  |
| -0.90 (-1.34,-0.45) | -0.63 (-1.00,-0.26) | -0.63 (-0.90,-0.36) | Control |

B

| ≥5 times/week |  |  |  |
| --- | --- | --- | --- |
| -0.91 (-1.82,0.01) | 1–2 times/week |  |  |
| -1.09 (-2.05,-0.12) | -0.18 (-0.99,0.63) | 3–4 times/week |  |
| -1.40 (-2.15,-0.65) | -0.49 (-1.02,0.03) | -0.31 (-0.93,0.30) | Control |

C

| 3–4 times/week |  |  |  |
| --- | --- | --- | --- |
| 0.91 (-0.67,2.49) | 1–2 times/week |  |  |
| 2.02 (0.92,3.12) | 1.11 (-0.03,2.25) | Control |  |
| 2.52 (-0.60,5.64) | 1.61 (-1.53,4.75) | 0.50 (-2.42,3.42) | ≥5 times/week |

Figure 23 League table of the effects of physical activity intervention frequencies on (A) social communication, (B) stereotyped behaviors, and (C) motor skills

*Notes*. For social communication and stereotyped behaviors, lower SMD values indicate better intervention effects, whereas for motor skills, higher SMD values indicate better intervention effects.

A

| ≤45 minutes |  |  |  |
| --- | --- | --- | --- |
| -0.04 (-0.46,0.37) | 46–60 minutes |  |  |
| -0.09 (-0.71,0.53) | -0.05 (-0.71,0.62) | >60 minutes |  |
| -0.67 (-0.91,-0.43) | -0.62 (-0.97,-0.28) | -0.58 (-1.15,-0.01) | Control |

B

| ≤45 minutes |  |  |
| --- | --- | --- |
| -0.33 (-1.11,0.46) | 46–60 minutes |  |
| -0.60 (-1.10,-0.10) | -0.27 (-0.88,0.33) | Control |

C

| >60 minutes |  |  |  |
| --- | --- | --- | --- |
| 2.80 (-0.24,5.84) | 46–60 minutes |  |  |
| 3.69 (0.64,6.74) | 0.89 (-0.39,2.17) | ≤45 minutes |  |
| 4.35 (1.44,7.26) | 1.55 (0.66,2.44) | 0.66 (-0.26,1.58) | Control |

Figure 24 League table of the effects of physical activity intervention session durations on (A) social communication, (B) stereotyped behaviors, and (C) motor skills

*Notes*. For social communication and stereotyped behaviors, lower SMD values indicate better intervention effects, whereas for motor skills, higher SMD values indicate better intervention effects.

A

| ＞12 weeks |  |  |  |
| --- | --- | --- | --- |
| -0.41 (-0.71,-0.11) | 9–12 weeks |  |  |
| -0.93 (-1.18,-0.68) | -0.52 (-0.69,-0.36) | Control |  |
| -1.04 (-1.87,-0.21) | -0.63 (-1.44,0.18) | -0.11 (-0.90,0.68) | ≤8 weeks |

B

| ＞12 weeks |  |  |  |
| --- | --- | --- | --- |
| -0.53 (-1.80,0.74) | 9–12 weeks |  |  |
| -0.71 (-2.25,0.83) | -0.18 (-1.30,0.93) | ≤8 weeks |  |
| -0.85 (-2.02,0.32) | -0.32 (-0.82,0.17) | -0.14 (-1.14,0.86) | Control |

C

| ≤8 weeks |  |  |  |
| --- | --- | --- | --- |
| 1.24 (-0.50,2.98) | 9–12 weeks |  |  |
| 2.01 (-0.22,4.24) | 0.77 (-1.09,2.63) | ＞12 weeks |  |
| 2.41 (0.90,3.91) | 1.17 (0.29,2.04) | 0.40 (-1.24,2.04) | Control |

Figure 25 League table of the effects of physical activity intervention periods on (A) social communication, (B) stereotyped behaviors, and (C) motor skills

*Notes*. For social communication and stereotyped behaviors, lower SMD values indicate better intervention effects, whereas for motor skills, higher SMD values indicate better intervention effects.

Table 6 The rankings of social communication, stereotyped behaviors, and motor skills for different physical activity frequencies, session durations, and periods

|  | Social communication | | |  | Stereotyped behaviors | | |  | Motor skills | | |
| --- | --- | --- | --- | --- | --- | --- | --- | --- | --- | --- | --- |
|  | SUCRA (%) | PrBest(%) | Mean rank |  | SUCRA (%) | PrBest(%) | Mean rank |  | SUCRA (%) | PrBest(%) | Mean rank |
| Frequency | | | | | | | | | | | |
| Control | 0.0 | 0.0 | 4.0 |  | 6.8 | 0.0 | 3.8 |  | 22.1 | 0.0 | 3.3 |
| 1–2 times/week | 55.4 | 11.1 | 2.3 |  | 55.7 | 2.3 | 2.3 |  | 64.6 | 11.4 | 2.1 |
| 3–4 times/week | 88.3 | 74.1 | 1.4 |  | 38.9 | 1.2 | 2.8 |  | 94.1 | 83.7 | 1.2 |
| ≥5 times/week | 56.3 | 14.8 | 2.3 |  | 98.7 | 96.5 | 1.0 |  | 19.2 | 4.8 | 3.4 |
| Session duration/minutes | | | | | | | | | | | |
| Control | 0.8 | 0.0 | 4.0 |  | 9.8 | 0.2 | 2.8 |  | 2.9 | 0.0 | 3.9 |
| ≤45 minutes | 72.9 | 38.3 | 1.8 |  | 89.3 | 79.3 | 1.2 |  | 33.8 | 0.3 | 3.0 |
| 46–60 minutes | 65.7 | 29.9 | 2.0 |  | 51.0 | 20.5 | 2.0 |  | 65.3 | 4.2 | 2.0 |
| ＞60 minutes | 60.6 | 31.8 | 2.2 |  | - | - | - |  | 98.0 | 95.5 | 1.1 |
| Period/week | | | | | | | | | | | |
| Control | 19.9 | 0.0 | 3.4 |  | 19.1 | 0.4 | 3.4 |  | 10.8 | 0.0 | 3.7 |
| ≤8 weeks | 15.8 | 0.6 | 3.5 |  | 39.1 | 13.2 | 2.8 |  | 95.9 | 89.5 | 1.1 |
| 9–12 weeks | 64.7 | 0.4 | 2.1 |  | 57.9 | 15.0 | 2.3 |  | 62.1 | 7.5 | 2.1 |
| ＞12 weeks | 99.7 | 99.0 | 1.0 |  | 83.9 | 71.4 | 1.5 |  | 31.2 | 3.0 | 3.1 |

*Notes*. Higher SUCRA values and lower mean ranks indicate better-performing physical activity intervention. Probability of it being the best physical activity intervention (PrBest);-

**Appendix 7** **PRISMA 2020 Checklist**

| **Section and Topic** | **Item #** | **Checklist item** | **Location where item**  **is reported** |
| --- | --- | --- | --- |
| **TITLE** | | |  |
| Title | 1 | The report is Identify as a meta-analysis and network meta-analysis systematic review. | 1 |
| **ABSTRACT** | | |  |
| Abstract | 2 | Summary contains all information in the PRISMA 2020 for Abstracts checklist, This study has been registered on the official Prospero website, and the registration code is CRD42023398221. | 1-3 |
| **INTRODUCTION** | | |  |
| Rationale | 3 | Described in the introduction. | 4-7 |
| Objectives | 4 | Described in the introduction. | 7 |
| **METHODS** | | |  |
| Eligibility criteria | 5 | Described in the methods (2.2). (Specify the inclusion and exclusion criteria for the review based on the PICOS principles.) | 8-9 |
| Information sources | 6 | Described in the methods(2.1). (Specify all databases, registers, websites, organizations, reference lists and other sources searched or consulted to identify studies. Specify the date when each source was last searched or consulted.) | 7-8 |
| Search strategy | 7 | Described in the methods(2.1).(Present the full search strategies for all databases, registers and websites, including any filters and limits used.) | 8 |
| Selection process | 8 | Described in the methods(2.1).( the methods used to decide whether a study met the inclusion criteria of the review, including how many reviewers screened each record and each report retrieved, whether they worked independently, and if applicable, details of automation tools used in the process.) | 10 |
| Data collection process | 9 | Described in the methods(2.1).(Specify the methods used to collect data from reports, including how many reviewers collected data from each report, whether they worked independently, any processes for obtaining or confirming data from study investigators, and if applicable, details of automation tools used in the process) | 10 |
| Data items | 10a | Described in the methods(2.4). (List and define all outcomes for which data were sought. Specify whether all results that were compatible with each outcome domain in each study were sought (e.g. for all measures, time points, analyses), and if not, the methods used to decide which results to collect.) | 8 |
|  | 10b | Described in the methods(2.3). (List and define all other variables for which data were sought (e.g. participant and intervention characteristics, funding sources). Describe any assumptions made about any missing or unclear information.) | 8 |
| Study risk of bias assessment | 11 | Described in the methods(2.3). (Specify the methods used to assess risk of bias in the included studies, including details of the tool(s) used, how many reviewers assessed each study and whether they worked independently, and if applicable, details of automation tools used in the process.) | 10-11 |
| Effect measures | 12 | Described in the methods(2.3). (Specify for each outcome the effect measure(s) (e.g. risk ratio, mean difference) used in the synthesis or presentation of results.) | 10-11 |
| Synthesis methods | 13a | Described in the methods(2.4). (Describe the processes used to decide which studies were eligible for each synthesis (e.g. tabulating the study intervention characteristics and comparing against the planned groups for each synthesis (item #5)).) | 10 |
|  | 13b | Described in the methods(2.4). (Describe any methods required to prepare the data for presentation or synthesis, such as handling of missing summary statistics, or data conversions.) | 10-11 |
|  | 13c | Described in the methods(2.4). (Describe any methods used to tabulate or visually display results of individual studies and syntheses.) | 10 |
|  | 13d | Described in the methods(2.4). (Describe any methods used to synthesize results and provide a rationale for the choice(s). If meta-analysis was performed, describe the model(s), method(s) to identify the presence and extent of statistical heterogeneity, and software package(s) used.) | 10-11 |
|  | 13e | Described in the methods(2.4). (Describe any methods used to explore possible causes of heterogeneity among study results (e.g. subgroup analysis, meta-regression).) | 10-11 |
|  | 13f | Described in the methods(2.4).（Describe any sensitivity analyses conducted to assess robustness of the synthesized results） | 10-11 |
| Reporting bias assessment | 14 | Described in the methods(2.4). (Describe any methods used to assess risk of bias due to missing results in a synthesis (arising from reporting biases).) | 9-10 |

**PRISMA 2020 Checklist**

| **Section and Topic** | **Item #** | **Checklist item** | **Location where item**  **is reported** |
| --- | --- | --- | --- |
| Certainty assessment | 15 | Described in the methods(2.4).(Describe any methods used to assess certainty (or confidence) in the body of evidence for an outcome.) | 9-10 |
| **RESULTS** | | |  |
| Study selection | 16a | Described in the results(3.1). (The results of the search and selection process, from the number of records identified in the search to the number of studies included in the review, using a flow diagram.) | 12,14 |
|  | 16b | Described in the results(3.1). (Cite studies that might appear to meet the inclusion criteria, but which were excluded, and explain why they were excluded.) | 14 |
| Study characteristics | 17 | Described in the results(3.1). ( Included study and present its characteristics.) | 12-13 |
| Risk of bias in studies | 18 | Described in the results(3.2). (Present assessments of risk of bias for each included study.) | 13 |
| Results of individual studies | 19 | Described in the results(3.3)(3.4). (For all outcomes, present, for each study: (a) summary statistics for each group (where appropriate) and (b) an effect estimate and its precision (e.g. confidence/credible interval), ideally using structured tables or plots.) | 15-21 |
| Results of syntheses | 20a | Described in the results(3.3). (For each synthesis, briefly summaries the characteristics and risk of bias among contributing studies.) | 15-20 |
|  | 20b | Described in the results(3.1). (The results of all statistical syntheses conducted. If meta-analysis was done, present for each the summary estimate and its precision (e.g. confidence/credible interval) and measures of statistical heterogeneity. If comparing groups, describe the direction of the effect.) | 15-21 |
|  | 20c | Described in the results(3.1)(3.4). (The results of all investigations of possible causes of heterogeneity among study results.) | 15-21 |
|  | 20d | Described in the results(3.5) （Present results of all sensitivity analyses conducted to assess the robustness of the synthesized results.） | 15-21 |
| Reporting biases | 21 | Described in the results(3.6). (The assessments of risk of bias due to missing results (arising from reporting biases) for each synthesis assessed.) | 13 |
| Certainty of evidence | 22 | Described in the results(3.6). (The assessments of certainty (or confidence) in the body of evidence for each outcome assessed.) | 15-21 |
| **DISCUSSION** | | |  |
| Discussion | 23a | Described in the discussion. (A general interpretation of the results in the context of other evidence.) | 22-30 |
|  | 23b | Described in the discussion. (Discuss any limitations of the evidence included in the review.) | 28-29 |
|  | 23c | Described in the discussion. (Discuss any limitations of the review processes used.) | 28-29 |
|  | 23d | Described in the discussion. (Discuss implications of the results for practice, policy, and future research.) | 28 |
| **OTHER INFORMATION** | | |  |
| Registration and protocol | 24a | This system overview has been recently submitted on the official PROSPERO website, and the registration results are still under review. | 7 |
|  | 24b | Described in the Support information. The review protocol can be accessed.( https://www.crd.york.ac.uk/prospero/#recordDetails) | 7 |
|  | 24c | Described in the Support information. No corrections have been made to the information provided in the registration or review protocol | 7 |
| Support | 25 | Described in the Acknowledgements. | 31 |
| Competing interests | 26 | Explained in the Acknowledgements. (No conflict of interest.) | 31 |
| Availability of data, code and other materials | 27 | Described in the Support information. Upload as a file to the support file.(Report which of the following are publicly available and where they can be found: template data collection forms; data extracted from included studies; data used for all analyses; analytic code; any other materials used in the review.) | 31 |

*From:* Page MJ, McKenzie JE, Bossuyt PM, et al. The PRISMA 2020 statement: an updated guideline for reporting systematic reviews. *BMJ* 2021;372:n71. doi:10.1136/bmj.n71

For more information, visit: **[www.prisma-statement.org](http://www.prisma-statement.org/)**[.](http://www.prisma-statement.org/)
